# Physiotherapy Rehabilitation in an Above-Knee Amputee Following Compartment Syndrome in Post-tibial Plateau Fracture: A Case Report

**DOI:** 10.7759/cureus.32855

**Published:** 2022-12-23

**Authors:** Deepiksha Chouhan, Pratik Phansopkar, Neha V Chitale, Madhu Lakhwani

**Affiliations:** 1 Musculoskeletal Physiotherapy, Ravi Nair Physiotherapy College, Datta Meghe Institute of Medical Sciences, Wardha, IND

**Keywords:** rehabilitation, above-knee amputation, gangrene, compartment syndrome, proximal tibia fibula fracture

## Abstract

Proximal tibia fractures are generally open fractures resulting in life-threatening complications. There is an increase in the prevalence of compartment syndrome post-tibial fractures. Though fasciotomy and wound debridement is the choice of treatment in such conditions, amputation becomes a necessity when there is widespread muscle and tissue damage. Specifically, compartment syndrome involving the lower limb is distressing as its symptoms can be misleading at times resulting in delayed diagnosis and hence life-threatening complications. A 48-year-old male met with a road accident and presented to the hospital with complaints of pain and an open wound over the anterior aspect of the right lower leg 14 days ago. He was managed by Ilizarov external fixator. Later he developed compartment syndrome as a complication of tibial plateau fracture, for which decompressive fasciotomy was performed. But due to irreversible tissue loss and gangrene, he had to undergo above-knee amputation followed by physiotherapy rehabilitation. We mainly focused on postoperative/pre-prosthetic rehabilitation. During postoperative rehabilitation, we concentrated on reducing phantom limb pain, preventing complications, and improving strength and endurance. Current literature claims that mirror therapy is effective in reducing phantom limb pain in post amputees, but there are only a few case reports emphasizing mirror therapy in particularly lower limb amputees. Therefore, we emphasized using mirror therapy for phantom limb pain in this case of lower limb amputation. It resulted in positive outcomes. Our broader aim was to strengthen the upper limbs and the intact lower limb so that the patient’s overall functional independence can be enhanced. Further prosthetic rehabilitation was planned in which we focused on gait and balance training. Physiotherapy rehabilitation improved the patient’s quality of life and independence.

## Introduction

Proximal tibia fibula fractures are one of the commonest causes of hospital admissions. In a study road, traffic accidents (RTA) were found to be the most prevalent cause and constituted about 78% of fractures, with nearly half of these involving motorbikes (42%). Young adults are most often afflicted by these fractures [1]. High-energy trauma is the major cause in young individuals while low-energy trauma is common in the elderly population. In developing countries, RTA is a growing concern due to increased morbidity and mortality rate. Other risk factors include osteoporosis, repetitive strain injuries, falls, and contact sports.

Severe complications of tibial plateau fractures, like popliteal artery injury, compartment syndrome, gangrene, infections, osteomyelitis, and amputation, may lead to life-threatening situations. Injury to the popliteal artery and compartment syndrome may exacerbate the condition of already dislocated tibial shaft fractures. The prognosis of this compound fracture is usually poor due to soft tissue involvement. 9% of open tibial fractures were associated with compartment syndrome [2]. Though compartment syndrome can be treated with an immediate fasciotomy procedure in cases where tissue and muscle loss are extensive, amputation becomes a necessity.

Amputation is the surgical removal of an extremity or part of an extremity as a result of an underlying condition or trauma. It is the last step in modern medicine when preserving a limb is virtually impossible. Amputation is broadly classified into two types - open and closed amputation. Peripheral vascular disease, neuropathy, nerve injuries, extreme heat and cold burns, diabetes mellitus, and trauma are the most frequent causes of amputation. The degree of amputation is determined by the vitality of the soft tissues present [3]. Loss of a limb has significant negative psychological, emotional, and economic implications on the individual as well as on his or her social domain [4].

Physiotherapy plays a crucial role in improving the quality of life in post amputees. Phantom limb pain is the prevalent condition in almost 50%-85% of amputees [5]. Mirror therapy is found to be an effective treatment in relieving phantom limb pain [6]. The role of physiotherapy is right from the early post-operative stage to the prosthetics stage. Strengthening and gait and balance training are the important aspects focused on during physiotherapy rehabilitation. On a broader aspect, physiotherapy aims at improving patient independence, and as a result, improving their social and professional life too.

## Case presentation

A 48-year-old male patient reported to the casualty with a complaint of pain and a wound over the anterior aspect just below the knee. The patient also complained of an inability to bear weight on the affected extremity. The patient had a history of a road traffic accident, where he collided with a four-wheeler while riding his bike back to home. He sustained an injury over the right lower leg. The patient was admitted and immediately advised an X-ray of the right hip and lower leg which revealed a compound grade III fracture of the proximal tibia and fibula. The patient underwent external fixation with the Ilizarov ring fixator later on the same day. Later, on physical evaluation, it was found that the distal pulsation at dorsalis pedis artery was absent. Also, the patient complained of pain and numbness along the right lower leg. Thus, the patient was advised color doppler scan and compartment pressure testing, which showed evidence of raised pressure and reduced flow in the anterior and lateral compartments, indicative of compartment syndrome. Fasciotomy and wound debridement procedures were performed for the same. Since the injury was severe, even after the fasciotomy the structures around the lateral and anterior compartments were not repaired efficiently. Hence, due to the decreased vascularity, the condition worsened and resulted in gangrene. The gangrene spread rapidly up to the thigh, due to this reason the patient underwent above-knee amputation of the right leg. The patient was then referred to the physiotherapy department for further management.

Clinical findings 

**Figure 1:**
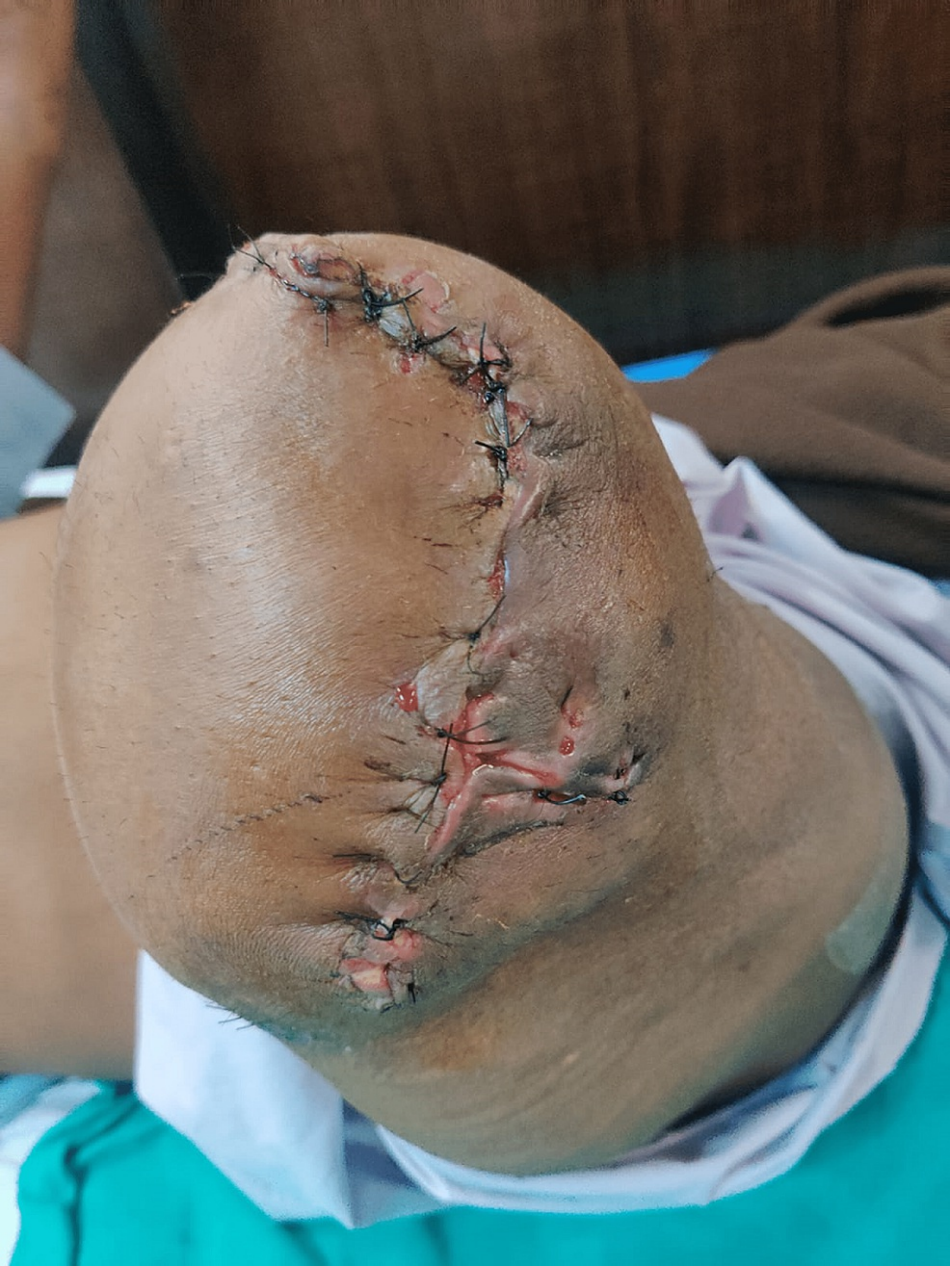
Stump end with fishmouth incision.

A physical evaluation was performed with the prior consent of the patient on postoperative day 4. The patient was conscious and well-oriented. All the vitals were normal. The patient was examined in a supine lying position with the head in neutral and the back supported properly. Both the anterior superior iliac spine (ASIS) were at the same level. The right leg was abducted and externally rotated. A semi-rigid dressing was present to prevent edema and promote wound healing. No significant muscle wasting was visible. Diffuse swelling was present around the stump. A fishmouth incision was visible at the operated site. The stump was conical in shape. No discharge was seen from the suture site, and healthy healing of the scar was present. The patient complained about throbbing pain at the amputated limb, as if the limb was still intact (Figure 1).

On Palpation

Grade 3 tenderness was present at the stump end. The local temperature was slightly raised. Sensations and circulation were intact. The stump length was measured to be 26 cm from the greater trochanter. The scar was non-adherent, with grade 3 tenderness. The length of the scar was 17 cm.

On Examination

Muscle wasting at the quadriceps was confirmed during girth measurement.

The pain was severe at the stump site on postoperative day 2. Even though the patient was on medications, the pain was rated as 7/10 on activity and 5/10 at rest on Numerical Pain Rating Scale. Further findings in range of motion (ROM) assessment are mentioned in Table 1.

**Table 1:** Range of motion: in postoperative assessment before rehabilitation.

Joint movement		Active range of motion		Passive range of motion	
		Right (amputated)	Left	Right	Left
Hip	Flexion	0º-70º	0º-110º	0º-75º	0-115
	Abduction	0º-40º	0º-45º	0º-45º	0º-47º
Knee	Flexion	NA	0º-130º	NA	0º-133º
Ankle	Planterflexion	NA	0º-40º	NA	0º-42º
	Dorsiflexion	NA	0º-20º	NA	0º-21º

On examination, the strength of the right hip musculature was reduced, and the left side strength was 4/5 due to the sedentary lifestyle of the patient (Table 2).

**Table 2:** Manual muscle testing (MMT) assessed according to MRC grading. MRC grading: Medical Research Council muscle strength grading

Muscle group		Right	Left
Hip	Flexors	2/5	4/5
	Extensors	2/5	4/5
	Internal rotators	2/5	4/5
	External rotators	2/5	4/5
Knee	Flexors	NT	4/5
	Extensors	NT	4/5
Ankle	Planterflexors	NT	4/5
	Dorsiflexors	NT	4/5

Physiotherapy management

**Figure 2:**
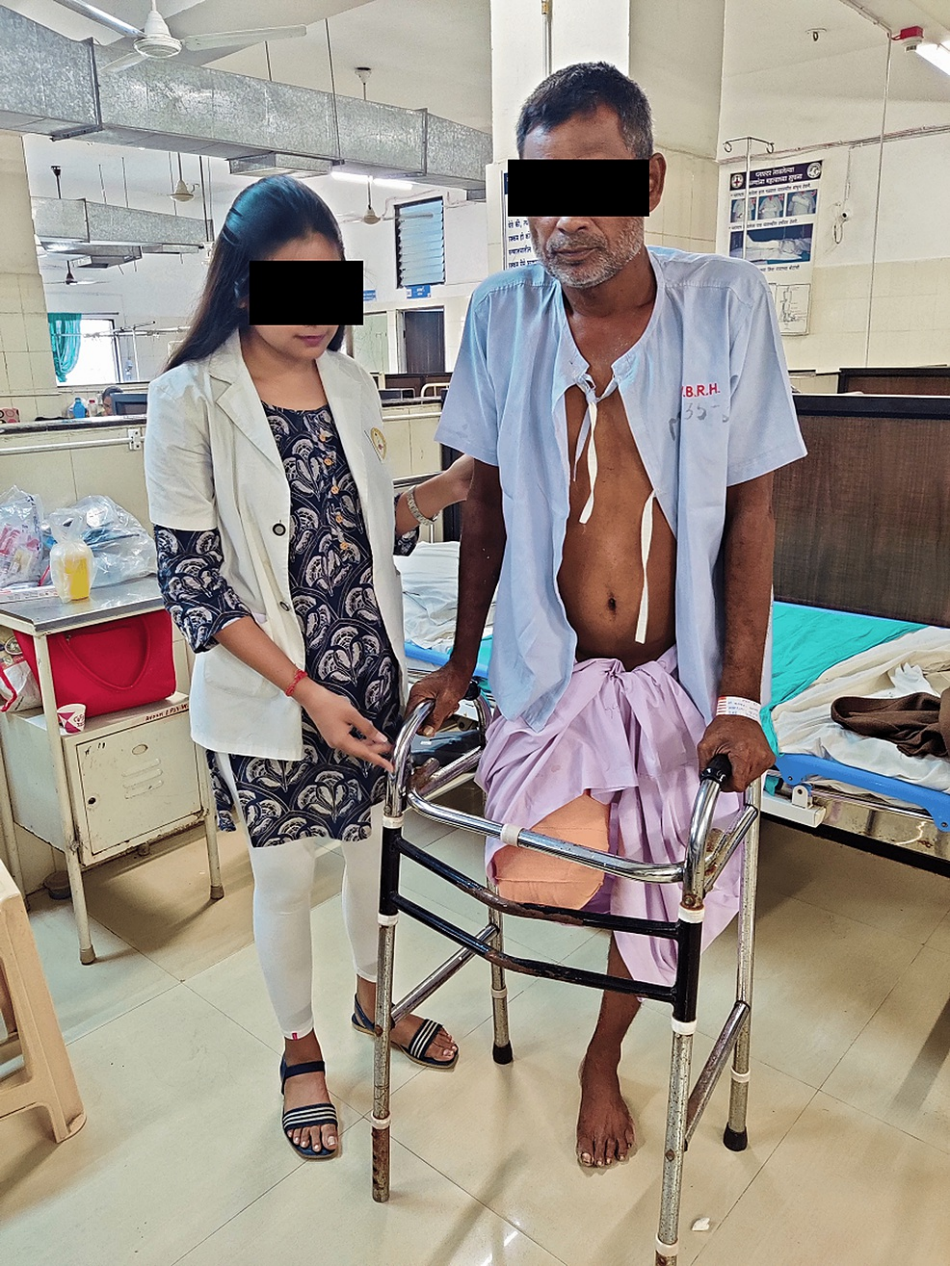
Ambulation using a walker.

Physiotherapy rehabilitation starts just after the amputation as early postoperative treatment or pre-prosthetic stage extending to the prosthetic stage which involves complete rehabilitation with prosthesis. During early rehabilitation, the patient was trained to ambulate using a walker (Figure 2). A structured postoperative and prosthetic rehabilitation protocol is mentioned in Tables 3 and Table 4, respectively. For reducing the phantom limb pain, we used mirror therapy for four weeks, which gave us positive outcomes along with transcutaneous electrical nerve stimulation (TENS). For strength and endurance training, progressive resistive exercises were included. balance training was performed on a therapy ball and parallel bar. After five weeks, a thorough assessment was done before prosthesis prescription - the patient had gained enough strength and was able to walk independently using a walker. He was then referred to the occupation therapist who advised him a lightweight above-knee prosthesis with an adjustable socket. It was an endoskeletal prosthesis, which requires less energy expenditure and provides good stability [7]. After six weeks, we started prosthetic rehabilitation, where we focused more on balance and gait training. 

**Table 3:** Postoperative rehabilitation (pre-prosthetic rehabilitation) reps: repetitions

	Goals	Physiotherapy management
Week 1 - 2	Patient education.	We have educated the patient about the condition, the importance of physiotherapy, and the impact of amputation on day-to-day life.
	To reduce phantom limb pain.	Transcutaneous electrical nerve stimulation (TENS) and massage were administered to relieve phantom limb pain. Low-intensity, high-frequency TENS was applied for 10 minutes twice a day. Massage technique including kneading and stroking was used to subside pain: twice a day for 15 minutes. Mirror therapy to reduce phantom limb pain was administered. A mirror was placed longitudinally and the patient was asked to position his left limb in front of the mirror. These created an illusion that patient’s both the limbs are intact. The patient moved his intact limb actively while watching in the mirror, which promoted relaxation and helped in reducing neuropathic pain: 6 days a week for 4 weeks.
	To prevent chest complication.	Deep breathing exercises including thoracic expansion exercises were taught to improve breathing: 7 reps thrice a day.
	To prevent stump edema	Limb was elevated and a semi-rigid dressing was done around the stump to reduce stump edema.
	To prevent scar adherence	Transverse friction massage for scar mobilization: 10 min twice a day.
	To prevent contracture.	Changing positions frequently in every two hours was advised. Prone positioning to prevent flexion contracture: 20 min thrice a day. Stretching of amputated limb musculature: 10 reps twice a day.
	To prevent circulatory complications.	Ankle toe movements and heel slides. Static glutes bilaterally: 10 reps thrice a day.
	To improve mobility of all joints.	Active range of motion exercises for upper extremity and lower extremity. Trunk mobility exercises. Pelvic bridging and pelvic tilting exercises. Mobility exercises of hip joint of the right side: 10 reps thrice a day.
	Bed mobility	Bed mobility including rolling, pushups, and transfers using upper extremity: thrice a day.
	To improve flexibility	Stretching exercises for hamstring and adductors of right: 10 reps thrice a day.
	To improve upper extremity strength.	Upper limb strengthening exercises using 1kg weight cuff: 10 reps thrice a day.
	To improve strength of lower extremity.	Hip flexors, adductors, abductors, and extensors strengthening using 1kg weight cuffs on the right side: 10 reps thrice a day.
	Transfer training.	Bed to chair transfer, wheelchair transfer.
	Early ambulation.	Room Ambulation using walker: thrice a day.
Week 2 - 6		The above-mentioned protocol was continued along-with following added exercises:
	To maintain and improve mobility.	Active range of motion exercises for upper extremity and lower extremity. Trunk mobility exercises. Pelvic bridging and pelvic tilting exercises. Mobility exercises of hip joint of the right side: 10 reps thrice a day.
	To improve and maintain muscle strength and endurance.	Progressive resistive exercises to the upper extremity and left lower extremity using 2kg weight cuff. Strengthening of hip flexor, extensor, adductor and abductor of the right extremity using theraband. Core strengthening exercises: 10 reps 3 times a day.
	To regain functional independence.	Teach ergonomics and activities of daily living, like dressing, grooming, toileting.
	Balance training	Static and dynamic balance training with therapy ball and parallel bar: thrice a day.
	Gait training	Gait training on a parallel bar to prepare the patient before application of prosthesis. Gait training using walker: thrice a day.

**Table 4:** Prosthetic rehabilitation reps: repetitions

Weeks	Goals	Physiotherapy management	Treatment regimen
Week 7 - 12	Strength training and endurance.	Progressive resisted exercises were continued as mentioned above.	10 reps twice a day, the intensity was progressed with time.
	Gait training.	Gait training on a parallel bar with prosthesis on. Progressing to walking using a cane.	
	Balance training.	Standing between a parallel bar and then weight shifting on the prosthetic limb. Weight shifting from side to side while standing against the table. Forward-backward weight shifting. Step-up and step-down while standing between parallel bars for support. Walking sideways Tandem standing. Single leg standing. Tandem walking. Walking around the obstacles. Stepping over the obstacles. Weight shifting training on balance board Stair climbing.	

Home Program Following Three Months of Physiotherapy Rehabilitation

Prosthetic training exercises were continued with progression. Timely follow-up should be considered by the patient to analyze the progress. Pre-rehabilitation and post-rehabilitation assessments were compared and are mentioned in Table 5.

**Table 5:** Pre- and post-rehabilitation assessment NPRS: Numerical Pain Rating Scale; MMT: Manual Muscle Testing; ROM: Range of Motion; NA: Not Applicable

Outcome measures	Pre-physiotherapy rehabilitation score		Post-physiotherapy rehabilitation score	
NPRS	7/10		0/10	
Right hip ROM	Active	Passive	Active	Passive
Flexion	0º-70º	0º-75º	0º-115º	0º-120º
Abduction	0º-40º	0º-45º	0º-45º	0º-47º
MMT for hip musculature:	Right		Right	
Flexors	2/5		5/5	
Abductors	2/5		5/5	
Internal rotators	2/5		5/5	
External rotators	2/5		5/5	
Barthel Index of activities of daily living	10/20		20/20	

## Discussion

In this case, the patient developed compartment syndrome as a complication of an open tibial fracture. A fasciotomy procedure was done to correct the effects of compartment syndrome, but it was not effective due to the severity of the condition. As a result, gangrene developed, for which an above-knee amputation procedure was performed. We planned a three-month physiotherapy protocol, which was broadly divided into two stages: the early postoperative stage and the prosthetic stage. During the early postoperative stage, we focused on improving muscle strength, reducing phantom limb pain, preventing complications, maintaining range of motion, endurance, and functional independence. In the prosthetic stage, our aim was to train balance and gait.

Phantom limb pain is the most prevalent postoperative complication in amputees. Various interventions have been discovered to treat phantom limb pain. Mirror therapy is one of the trending physiotherapy approaches for managing phantom limb pain and sensations [6]. It is found to be an effective approach, although there is less literature emphasizing its effectiveness and use in lower limb amputation. In our case, we used mirror therapy, in which the intact limb was placed in front of the mirror and the patient was asked to move his intact limb actively so that it created an illusion that the patient’s both limbs is intact. This worked as a placebo effect and helped in promoting relaxation and a sense of pain relief. We also used TENS for pain relief, which proved to be effective in our case. As inferred from evident literature, it was concluded that phantom limb pain was significantly reduced when conventional physiotherapy adjuncts were administered along with mirror therapy in prosthesis users [8].

Balance and postural training after amputation have a key role in helping the amputee to regain their independence post-amputation. Various studies have been conducted to learn about recent advances in such cases. In 2012, a study was held that investigated the effect of rehabilitation using video games on balance training in children and adolescents with lower limb amputation - a positive result was found, but in the context of long-term results, it was quite unclear [9]. In our case, we used the conventional physiotherapy approach for balance and gait training, which helped in improving the overall functional independence of the patient.

A study performed in 2007 declared that short and intensive physiotherapy rehabilitation gives positive results in terms of improving walking speed and other functional activities [10].

Our main aim was to enhance the complete independence of the patient; hence, after the application of the prosthesis, the patient was trained for balance and gait. In a literature review published in 2018, it was concluded that the application of physiotherapy demonstrated beneficial effects on functional status. In a study, it was revealed that early use of physiotherapy rehabilitation and a suitable prosthesis was found to significantly improve functional outcomes. There was a reduction in energy usage, enhanced balance, and normalization of gait patterns [11].

In a study, it was found that patients fitted with prostheses had a better level of ability to perform activities of daily living (ADLs) as compared to those who did not use prostheses. They also demonstrated higher SF-12 mental and physical scores. These patients were shown to be more independent in ambulation [12]. In this case, there was a remarkable improvement in the patient’s functional activity after prosthetic rehabilitation. His confidence also leveled up.

## Conclusions

In the case presented, we concentrated more on pre-prosthetic rehabilitation by training residual limb and upper extremity for transfer and ambulation. For relieving phantom limb pain, mirror therapy was administered, which was found to be effective. Later, after the application of the prosthesis, we emphasized more on gait and balance training. As a result, the complete independence of the patient was enhanced.

## References

[1] Clelland SJ, Chauhan P, Mandari FN (2016). The epidemiology and management of tibia and fibula fractures at Kilimanjaro Christian Medical Centre (KCMC) in Northern Tanzania. Pan Afr Med J.

[2] Blick SS, Brumback RJ, Poka A, Burgess AR, Ebraheim NA (1986). Compartment syndrome in open tibial fractures. J Bone Jt Surg.

[3] Molina CS, Faulk J (2022 Jan-). Lower extremity amputation. StatPearls [Internet].

[4] Shankar P, Grewal VS, Agrawal S, Nair SV (2020). A study on quality of life among lower limb amputees at a tertiary prosthetic rehabilitation center. Med J Armed Forces India.

[5] Herrador Colmenero L, Perez Marmol JM, Martí-García C (2018). Effectiveness of mirror therapy, motor imagery, and virtual feedback on phantom limb pain following amputation: a systematic review. Prosthet Orthot Int.

[6] Wang F, Zhang R, Zhang J, Li D, Wang Y, Yang Y-H, Wei Q (2021). Effects of mirror therapy on phantom limb sensation and phantom limb pain in amputees: a systematic review and meta-analysis of randomized controlled trials. Clin Rehabil.

[7] Irons G, Mooney V, Putnam S, Quigley M (1977). A lightweight above-knee prosthesis with an adjustable socket. Orthot Prosth.

[8] Limakatso K, Parker R (2021). Treatment recommendations for phantom limb pain in people with amputations: an Expert Consensus Delphi Study. PM R.

[9] Andrysek J, Klejman S, Steinnagel B (2012). Preliminary evaluation of a commercially available videogame system as an adjunct therapeutic intervention for improving balance among children and adolescents with lower limb amputations. Arch Phys Med Rehabil.

[10] Rau B, Bonvin F, de Bie R (2007). Short-term effect of physiotherapy rehabilitation on functional performance of lower limb amputees. Prosthet Orthot Int.

[11] Ülger Ö, Yıldırım Şahan T, Çelik SE (2018). A systematic literature review of physiotherapy and rehabilitation approaches to lower-limb amputation. Physiother Theory Pract.

[12] Fedorka CJ, Chen AF, McGarry WM (2011). Functional ability after above-the-knee amputation for infected total knee arthroplasty. Clin Orthop Relat Res.

